# Genotypes and phenotypes of motor neuron disease: an update of the genetic landscape in Scotland

**DOI:** 10.1007/s00415-024-12450-w

**Published:** 2024-06-09

**Authors:** Danielle J. Leighton, Morad Ansari, Judith Newton, Elaine Cleary, Laura Stephenson, Emily Beswick, Javier Carod Artal, Richard Davenport, Callum Duncan, George H. Gorrie, Ian Morrison, Robert Swingler, Ian J. Deary, Mary Porteous, Siddharthan Chandran, Suvankar Pal, the Lothian Birth Cohorts Group, the CARE-MND Consortium, Andrew Bethell, Susan Byrne, Myles Connor, Gillian Craig, Ondrej Dolezal, Moira Flett, Louise Gardiner, Jessica Gill, Isaac Chau, Janice Hatrick, Micheala Johnson, Katja Lassak, Juan Larraz, Helen Lennox, Pauline MacDonald, Laura Marshall, Dympna McAleer, Alison McEleney, Kitty Millar, Louise Murrie, David Perry, Gowri Saravanan, David Simpson, Susan Stewart, Dorothy Storey, Gill Stott, David Thompson, Carol Thornton, Carolyn Webber, Michael Wong, Sarah Harris, James Prendergast, Tom Russ, Adele Taylor, Ian Deary

**Affiliations:** 1 School of Psychology & Neuroscience, University of Glasgow, Glasgow, UK; 2 The Euan MacDonald Centre for Motor Neuron Disease Research, University of Edinburgh, Edinburgh, UK; 3 Anne Rowling Regenerative Neurology Clinic, Royal Infirmary, Edinburgh, UK; 4 Centre for Clinical Brain Sciences, University of Edinburgh, Edinburgh, UK; 5 Institute of Neurological Sciences, Queen Elizabeth University Hospital, 1345 Govan Road, Glasgow, G51 4TF UK; 6 South East Scotland Genetics Service, Western General Hospital, Edinburgh, UK; 7 Department of Neurology, NHS Highland, Inverness, UK; 8 Department of Neurology, Aberdeen Royal Infirmary, Aberdeen, UK; 9 Department of Neurology, NHS Tayside, Dundee, UK; 10 Lothian Birth Cohorts Group, Department of Psychology, University of Edinburgh, Edinburgh, UK; 11 UK Dementia Research Institute, University of Edinburgh, Edinburgh, UK

**Keywords:** Motor neuron disease, Amyotrophic lateral sclerosis, Genetic epidemiology, Genotype-phenotype, SOD1, C9orf72

## Abstract

**Background:**

Using the Clinical Audit Research and Evaluation of Motor Neuron Disease (CARE-MND) database and the Scottish Regenerative Neurology Tissue Bank, we aimed to outline the genetic epidemiology and phenotypes of an incident cohort of people with MND (pwMND) to gain a realistic impression of the genetic landscape and genotype–phenotype associations.

**Methods:**

Phenotypic markers were identified from the CARE-MND platform. Sequence analysis of 48 genes was undertaken. Variants were classified using a structured evidence-based approach. Samples were also tested for *C9orf72* hexanucleotide expansions using repeat-prime PCR methodology.

**Results:**

339 pwMND donated a DNA sample: 44 (13.0%) fulfilled criteria for having a pathogenic variant/repeat expansion, 53.5% of those with a family history of MND and 9.3% of those without. The majority (30 (8.8%)) had a pathogenic *C9orf72* repeat expansion, including two with intermediate expansions. Having a *C9orf72* expansion was associated with a significantly lower Edinburgh Cognitive and Behavioural ALS Screen ALS-Specific score (*p* = 0.0005). The known pathogenic *SOD1* variant p.(Ile114Thr), frequently observed in the Scottish population, was detected in 9 (2.7%) of total cases but in 17.9% of familial cases. Rare variants were detected in *FUS* and *NEK1*. One individual carried both a *C9orf72* expansion and *SOD1* variant.

**Conclusions:**

Our results provide an accurate summary of MND demographics and genetic epidemiology. We recommend early genetic testing of people with cognitive impairment to ensure that *C9orf72* carriers are given the best opportunity for informed treatment planning. Scotland is enriched for the *SOD1* p.(Ile114Thr) variant and this has significant implications with regards to future genetically-targeted treatments.

**Supplementary Information:**

The online version contains supplementary material available at 10.1007/s00415-024-12450-w.

## Introduction

The genetics of motor neuron disease (MND) is an evolving landscape. People with MND (pwMND) are becoming increasingly aware of, and interested in, pursuing genetic testing. For those who proceed with genetic testing, interpretation of variant implications brings another dimension to their complex disease.

Classification of variant pathogenicity is problematic for many genetic diseases but becomes particularly difficult within the scope of a rare disease with multiple genetic links such as MND [1]. Barriers to firm classification have been acknowledged, including the relative paucity of functional studies and large pedigrees for assessment of co-segregation [2]. Variants of Uncertain Clinical Significance (VUS) are inevitable and bring their own diagnostic difficulties. There remains no consensus classification system for assessment of MND variant causality [3]. Latterly, the American College of Medical Genetics and Association for Molecular Pathology (ACMG-AMP) framework has been adopted [4–8].

The implications for MND are clear when we consider the imminent advent of genetically stratified therapies, which have the potential to involve prolonged commitment to invasive treatments. The recently Food and Drug Administration (FDA) approved drug, Tofersen, for MND mediated by the *SOD1* gene is currently under review by UK and European drug regulators [9]. Accurate description of the genetic epidemiology of MND in Scotland will be important for planning potential delivery of these treatments. On a more immediate level there is the concern of burdening patients and their relatives with the anxiety of uncertain future risk [10].

In a previous study of the genetic epidemiology of MND in Scotland, 17% of pwMND had a potential genetic cause of their disease using a limited gene panel [5]. Key genetic mutations in this population include *C9orf72* expansions and a Scottish founder variant in the *SOD1* gene (p.(Ile114Thr)) [5, 7, 11]. In 2014, a neurodegenerative gene panel comprising 11 genes was incorporated into clinical practice in Scotland. However, with the emergence of new discoveries regarding genetic associations in MND, this quickly became outdated. The burden of MND-associated rare genes in this population is unknown. This information is required to inform clinical and diagnostic testing, outline priorities for future disease modelling studies and identify families for whom genetically-targeted treatments may be an option.

Scotland benefits from a longstanding national register for pwMND, now hosted by the CARE-MND Platform (Clinical Audit Research and Evaluation for MND) [12]. A broad selection of variables are available to allow us to appreciate the phenotypes of pwMND.

We aimed to study the genetic epidemiology and phenotypes of a well-characterised incident population of pwMND in Scotland diagnosed between 2015 and 2017. This cohort was carefully studied at the inception of the CARE-MND platform and coincided with a boost in MND nursing care in Scotland [12, 13]. Using an inclusive and contemporary research gene panel and adopting stringent variant classification methods, we aimed to obtain a realistic representation of the clinical impact of genetics in the Scottish MND population and identify any genotype–phenotype associations. Findings would inform clinical gene-panel testing pathways.

## Methods

### Gene-panel selection

A review was undertaken in 2015–2016 to update the existing 11-gene neurodegenerative disease gene panel. Existing UK-based MND-related gene panels and resources were examined [14–19]. The final panel consisted of 49 MND-associated genes for research study: ALS2, ANG, ANXA11, APP, ATL1, BSCL2, CCNF, CHCHD10, CHMP2B, CSF1R, DAO, DCTN1, ERBB4, FIG4, FUS, GRN, hnRNPA1, hnRNPA2/BA, HTRA1, ITM2B, MAPT, MATR3, NEFH, NEK1, NIPA1, NOTCH3, OPTN, PFN1, PLP1, PRNP, PRPH, PSEN1, PSEN2, REEP1, SETX, SIGMAR1, SOD1, SPAST/SPG4, SPG11, SPG20, SQSTM1, TAF15, TARDBP, TBK1, TUBA4A, UBQLN2, VAPB, VCP and the C9orf72 repeat expansion (previously published, 10.1007/s00415-022-11505-0) [7].

### Recruitment and ethical approvals

All people diagnosed with MND in Scotland are invited to participate in the Scottish MND Register via the CARE-MND platform (ethical approvals MREC/98/0/56 1989–2010, 10/MRE00/78 2011–2015, and the Scotland A Research Ethics Committee 15/SS/0126 2015 onwards). DNA samples were donated to the Scottish MND DNA Bank and the Scottish Regenerative Neurology Tissue Bank (MREC/98/0/56 1989–2010, 10/MRE00/77 2011 to 2013, 13/ES/0126 2013–2015, 15/ES/0094 2015-present). The Lothian Birth Cohorts (LBC) – a research population of Scottish adults born in 1921 and 1936—were used as ancestry-matched genetic controls [20]. Ethical permission for the LBC1936 study protocol was obtained from the Multi-Centre Research Ethics Committee for Scotland (Wave 1: MREC/01/0/56), the Lothian Research Ethics Committee (Wave 1: LREC/2003/2/29), and the Scotland A Research Ethics Committee (Waves 2, 3,4 and 5: 07/MRE00/58). Ethical permission for the LBC1921 study protocol was obtained from the Lothian Research Ethics Committee (Wave 1: LREC/1998/4/183; Wave 2: LREC/2003/7/23; Wave 3: LREC1702/98/4/183), the Scotland A Research Ethics Committee (Waves 4 and 5: 10/MRE00/87).

### Genotyping

Samples were genotyped using QiaSeq Amplicon Sequencing. Sequence analysis of a panel of 48 genes causally associated with neurodegeneration was carried out using a custom-designed QIAseq assay for library construction as per manufacturer’s instructions (QIAGEN). In brief, 80 ng of DNA was fragmented followed by adaptor ligation. Target enrichment was carried out by single primer extension, followed by sample indexing and amplification. Equal volumes of libraries were combined, and quantified using a Quantus™ Fluorometer as per manufacturer’s instructions. Paired-end sequencing of the resulting DNA library (at a concentration of 10 pM) was performed using an Illumina MiSeq instrument. Alignment and variant calling was performed using the QIAGEN CLC Genomics Workbench as per in-house standard operating procedure. Sequence read coverage was assessed against a browser extensible data (BED) file containing the genomic regions of interest.

All samples were also screened for *C9orf72* hexanucleotide expansions using repeat-prime PCR methods [21], taking the total gene count to 49. Expansions > 30 repeats were considered pathogenic.

### Variant classification

Each variant was systematically reviewed using the ACMG-AMP 28-point system and adhering to the Association for Clinical Genomic Science (ACGS) UK 2020 guidelines [6, 22]. A modified Delphi approach[23, 24] was taken to outline consensus criteria for the major MND-associated genes. Classification criteria have been used by our group previously[7] and are detailed in Supplementary Material 1.

### Phenotyping and genotype–phenotype associations

Data were available for individuals who had provided written informed consent to data-sharing via the Scottish MND Register. **A **wide breadth of premorbid demographical (sex, ethnicity), environmental (smoking, heavy metal or pesticide exposure) and health-related variables (exercise, history of head injury, history of autoimmune disease, cardiovascular disease, malignancy, psychiatric illness), family history (of MND, dementia, early-onset dementia, other neurological disease, psychiatric disease) as well as markers of disease (age of onset, time to diagnosis, site of onset, classification of MND, riluzole use, feeding tube insertion, non-invasive ventilaton (NIV) use) were extracted and pre-processed from the CARE-MND database. MND-specific tools were used, including the validated and globally recognised Edinburgh Cognitive and Behavioural Amyotrophic Lateral Sclerosis Screen (ECAS)[25] and the revised Amyotrophic Lateral Sclerosis Functional Rating Scale (ALSFRS-R), a measure of limb, bulbar and respiratory function in daily living. Rate of ALSFRS-R decline was calculated using the concept of the ‘preslope’ or ALSFRS-R-based linear estimate of rare of disease progression, which is a recognised measurement in MND observational studies and clinical trials [26].

The following group were studied for genotype–phenotype associations: (i) pwMND with *C9orf72* expansions, (ii) pwMND with *SOD1* mutations, (iii) pwMND with *SOD1* p.(Ile114Thr) variants. Descriptive statistics for phenotypic variables by group were summarised. In view of the large number of variables included relative to the number of individuals studied, univariate statistics were used to determine significance.

### Statistical analyses

Data were formatted and analysed using R statistical programming [27]. Krippendorff’s alpha (k-alpha) statistic was used to assess formally inter-rater reliability; k-alpha score ranges from 0 (no concordance) to 1 (complete concordance) with good agreement considered ≥ 0.80 [28]. Univariate statistics (Fisher’s exact tests, t tests and Wilcoxon rank-sum tests) were used for association testing with correction for multiple testing using the Bonferroni method.

## Results

Of 619 people with MND diagnosed in Scotland in 2015–2017, 437 (70.6%) consented to share their medical record data via the MND Register. The number of DNA samples donated by incident pwMND 2015–2017 was 339; this is representative of 54.8% of the incident MND cohort 2015–17 [13].

### Variant classification

After three rounds of honing classification approaches using the modified Delphi method, classification concordance between raters was 84%, with an error rate of 3.0% and a mean k-alpha of 0.91 (95% CI 0.87, 0.95). In a review of use of the ACMG-AMP criteria amongst nine laboratories, average intra-laboratory k-alpha was 0.91 [29]. As our third-round k-alpha was ≥ 0.80 and compatible with clinical sequencing laboratory agreements, the consensus methods were considered to be appropriate for use.

### Genotyping: *C9orf72*

Repeat-prime PCR for the *C9orf72* expansion identified 29/339 (8.6%) individuals with > 30 GGGGCC repeats. Of these, one patient had an unusual intermediate-length expansion (70 repeats). One further patient had 28 repeats. Meta-analysis suggests that intermediate expansions 24–30 repeats in length are associated with MND [30]. In view of this evidence, our intermediate-length samples were both considered significant giving a final population frequency of 30/339 (8.8%).

### Genotyping: panel sequencing

On gene panel sequencing, depth of coverage (≥ 20X) was, on average, 98% across the regions of interest. After VarSeq variant filtering, 503 variants were identified in 339 samples. Variants (including benign variants and VUS) were identified in 278/339 (82.0%) of samples. Fifteen (15/339, 4.4%) had a variant meeting criteria for pathogenicity (Table 2). The number of pwMND with a VUS in an MND-associated gene was 88 (88/339, 25.9%). Of these, 38 individuals (38/339, 1.1%) had a VUS which met some pathogenic ACMG-AMP criteria (‘hot’ VUS). These are summarised in Supplementary Material 2. One patient had both a pathogenic missense variant and a pathogenic *C9orf72* expansion. A further six individuals had two variants of interest (including VUS meeting some criteria for pathogenicity) and these are summarised in Supplementary Material 3.

After the *C9orf72* expansion, the most common variant in this MND cohort was the *SOD1* p.(Ile114Thr) variant (*n* = 9), previously described as a founder mutation in the Scottish population [5, 7, 11]. Three further *SOD1* variants were identified. One of these, p.(Ala146Asp), had previously been seen in a different Scottish individual with MND[5] and was absent from the control population and gnomAD. Two other *SOD1* missense variants were observed: p.(Gln23His) and p.(Gly73Cys). These variants have not previously been identified in the Scottish population. The frequency of *SOD1* mutations in this cohort was therefore 12/339, 3.5%.

A loss of function (LoF) variant (p.Tyr479Metfs*50) was observed in the *FUS* gene. This is a novel variant in a genomic location near to previously described MND-associated frameshift mutations.

Two individuals were found to have a LoF variant in exon 21 of *NEK1*, which was absent from gnomAD and classified as pathogenic (p.(Glu634Lysfs*11)). As far as we can determine from patient histories, the patients were unrelated. A further LoF *NEK1* splice donor variant (NM_012224.2:c.868 + 1G > C) was identified in a different individual; this is predicted to abolish the canonical splice donor site and initiate nonsense-mediated decay. However, the variant is present in gnomAD and so it did not meet criteria for pathogenicity (see Supplementary Material 2).

**Fig. 1:**
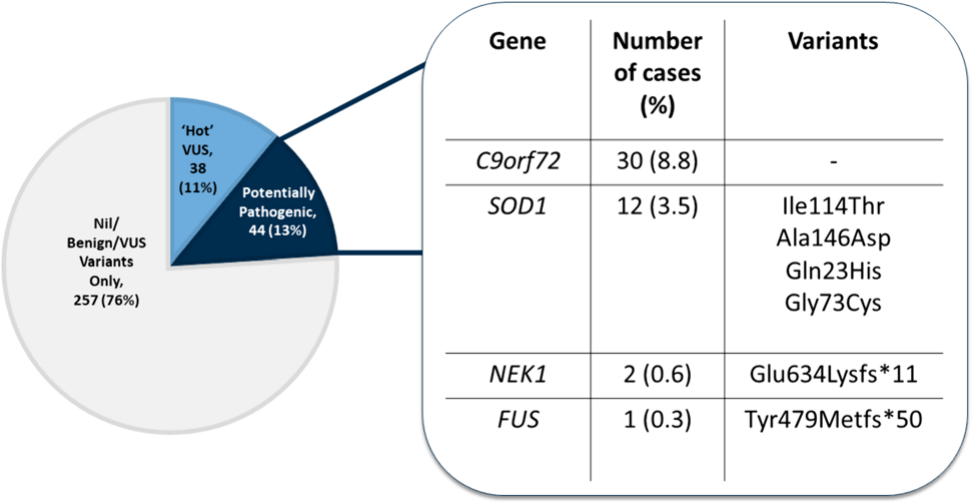
Summary of genetic epidemiology of incident MND cohort 2015–2017, *VUS* variant of uncertain clinical significance

In summary, a total of 44/339 (13.0%) of individuals (including those with *C9orf72* expansions) had a potential genetic explanation for their disease (Fig. 1) (see Table 1).

**Table 1:** Variants identified in incident MND cohort 2015–2017

Gene	Genomic position	Variant DNA change	Variant protein change	Variant type	Pathogenic classification	Number cases	Number controls
Variants meeting criteria for pathogenicity							
*SOD1*	21:33039672T > C	c.341T > C	p.Ile114Thr	Missense	Pathogenic	9	0
*SOD1*	21:33040863C > A	c.437C > A	p.Ala146Asp	Missense	Pathogenic	1	0
*SOD1*	21:33032151G > C	c.69G > C	p.Gln23His	Missense	Pathogenic	1	0
*SOD1*	21:33038809G > T	c.217G > T	p.Gly73Cys	Missense	Pathogenic	1	0
*FUS*	16:31202325 T > -	c.1435delT	p.Tyr479Metfs*50	LoF frameshift	Pathogenic	1	0
*NEK1*	4:170428210TC > -	c.1900_1901delGA	p.Glu634Lysfs*11	LoF frameshift	Pathogenic	2	0

*LoF* loss of function

### Genotype–phenotype associations

Phenotypic characteristics of the genotyped cohort (*n* = 339) as well as those of individuals with i) *C9orf72* pathogenic expansions, (ii) *SOD1* pathogenic variants and (iii) *SOD1* p(.Ile114Thr) variants are summarised in Table 2.

**Table 2:** Descriptive statistics of phenotypic characteristics of the total analysed cohort (*n* = 339) and by genotype group

Phenotypic characteristic	Missing data (%)	Summary statistic/test	All (n = 339)	*C9orf72* (n = 30)	*SOD1* inc. I114T (n = 12)	*SOD1* I114T (n = 9)
Sex	0	Male (%) Fisher’s	220 (64.9) –	15 (50.0) *p* = 0.1	9 (75.0) *p* = 0.6	6 (66.7) *p* = 1.0
Ethnicity	2.7	White Scottish (%) Fisher’s	326 (98.8) –	29 (100) *p* = 1.0	12 (100) *p* = 1.0	9 (100) *p* = 1.0
Ever smoked	7.7	Yes (%) Fisher’s	176 (56.2) –	14 (48.3) *p* = 0.4	6 (50.0) *p* = 0.8	4 (44.4) *p* = 0.5
Exercise participation	15.6	Median (IQR) Wilcoxon	Mod (Light–Mod) –	Mod (Light-Mod) *p* = 0.5	Mod (Mod-Heavy) *p* = 0.3	Mod (Mod-Heavy) *p* = 0.1
Heavy metal or pesticide exposure	28.0	Yes (%) Fisher’s	60 (24.6) –	5 (20.8) *p* = 0.8	2 (20.0) *p* = 1.0	2 (25.0) *p* = 1.0
PMH cardiovascular disease	0	Yes (%) Fisher’s	164 (48.4) –	12 (40.0) *p* = 0.3	6 (50.0) *p* = 1.0	4 (44.4) *p* = 1.0
PMH autoimmune disease	0	Yes (%) Fisher’s	53 (15.6) –	6 (20.0) *p* = 0.4	0 (0) *p* = 0.2	0 (0) *p* = 0.4
PMH malignancy	0	Yes (%) Fisher’s	28 (8.3) –	1 (3.3) *p* = 0.5	1 (8.3) *p* = 1.0	1 (11.1) *p* = 0.5
PMH psychiatric disease	0	Yes %) Fisher’s	71 (20.9) –	4 (13.3) *p* = 0.4	4 (33.3) *p* = 0.3	3 (33.3) *p* = 0.4
History of head injury	26.8	Yes (%)	67 (27.0) –	3 (13.0) *p* = 0.1	3 (33.3) *p* = 0.7	2 (28.6) *p* = 1.0
History of blood transfusion	29.8	Yes (%)	25 (10.5) –	0 (0) *p* = 0.1	1 (11.1) *p* = 1.0	1 (14.3) *p* = 0.5
Family history of MND	1.8	Yes (%) Fisher’s	28 (8.4) –	**9 (30.0)** *p* = 0.0002*	**6 (50.0)** *p* = 0.0001*	**5 (55.6)** *p* = 0.0003*
Family history of dementia	3.5	Yes (%) Fisher’s	97 (29.7) –	9 (30.0) *p* = 1.0	5 (45.5) *p* = 0.3	3 (37.5) *p* = 0.7
Family history of early-onset dementia	3.5	Yes (%) Fisher’s	15 (4.6) –	3 (10.0) *p* = 0.1	1 (9.1) *p* = 0.4	0 (0) *p* = 1.0
Family history of other neurological conditions	5.0	Yes (%) Fisher’s	94 (29.2) –	14 (46.7) *p* = 0.03	7 (63.6) *p* = 0.02	4 (50.0) *p* = 0.2
Family history of psychiatric conditions	11.5	Yes (%) Fisher’s	49 (16.3) –	7 (26.9) *p* = 0.2	2 (18.2) *p* = 0.7	0 (0) *p* = 0.4
Age of onset (years)	0.6	Mean (SD) *t*-test	63.1 (10.8) –	60.7 (7.8) *p* = 0.1	58.4 (7.8) *p* = 0.06	60.1 (6.5) *p* = 0.2
Time to diagnosis (months)		Median (IQR) Wilcoxon	12.0 (8.0–23.0) –	10.0 (7.0–18.0) *p* = 0.08	17.0 (8.5–35.8) *p* = 0.3	14.0 (7.0–35.0) *p* = 0.8
Site of onset	0	Bulbar % Limb (%) Other (%) Fisher’s	110 (32.5) 211 (62.2) 18 (5.3) –	11 (36.7) 15 (50.0) 4 (13.3) *p* = 0.08	1 (8.3) 11 (91.7) 0 (0) *p* = 0.1	0 (0) 9 (100) 0 (0) *p* = 0.07
Classification	0	ALS (%) MND-FTD (%) PLS (%) PMA (%) PBP (%) Bibrachial (%) Fisher’s	261 (77.0) 18 (5.3) 14 (4.1) 16 (4.7) 20 (5.9) 10 (3.0) –	23 (76.7) 5 (16.7) 0 (0) 0 (0) 2 (6.7) 0 (0) *p* = 0.08	11 (91.7) 0 (0) 0 (0) 0 (0) 0 (0) 1 (8.3) *p* = 0.7	9 (100) 0 (0) 0 (0) 0 (0) 0 (0) 0 (0) *p* = 1.0
ALSFRS-R Preslope	21.5	Median (IQR) Wilcoxon	0.58 (0.28–1.00) –	0.71 (0.32–1.11) *p* = 0.3	0.23 (0.18–0.58) *p* = 0.03	0.40 (0.14–0.62) *p* = 0.1
ECAS ALS specific score	44.5	Median (IQR) Wilcoxon	81.5 (70.8–87.0) –	**69.0 (55.8–79.0)** *p* = 0.0005*	86 (76.5–87.0) *p* = 0.4	87.0 (78.0–87.0) *p* = 0.4
ECAS ALS non-specific score	44.5	Median (IQR) Wilcoxon	28.0 (24.0–31.0) –	25.5 (24.0–29.0) *p* = 0.3	26.5 (26.0–30.5) *p* = 0.9	26.0 (25.0–30.0) *p* = 1.0
Riluzole use	0	Yes (%) Fisher’s	138 (40.7) –	14 (46.7) *p* = 0.6	5 (41.7) *p* = 1.0	3 (33.3) *p* = 0.7
Feeding tube inserted	0.3	Yes (%) Fisher’s	115 (34.0) –	10 (33.3) *p* = 1.0	0 (0) *p* = 0.01	0 (0) *p* = 0.03
NIV use	0.3	Yes (%) Fisher’s	99 (29.3) –	2 (6.7) *p* = 0.003	8 (66.7) *p* = 0.007	7 (77.8) *p* = 0.003

*PMH* Past Medical History, *ALS* Amyotrophic Lateral Sclerosis, *MND-FTD* Motor Neuron Disease with Frontotemporal Dementia, *PLS* Primary Lateral Sclerosis, *PMA* Progressive Muscular Atrophy, *PBP* Progressive Bulbar Palsy, *ALSFRS-R* Revised Amyotrophic Functional Rating Scale, *ECAS* Edinburgh Cognitive and Behavioural ALS Screen, *NIV* Non-invasive ventilation

Bonferroni-corrected significant values are highlighted in bold and starred (*) (Bonferroni-corrected *p* = 0.0019)

Univariate statistics with Bonferroni correction revealed that a family history of MND and lower ECAS: ALS-Specific Score were associated with having a *C9orf72* expansion (Table 2). There were two individuals with intermediate-length repeat expansions. Both individuals were diagnosed in their early sixties with upper limb-onset disease and had diagnoses of ALS with cognitive impairment identified via ECAS measurements (ECAS total score 73 in patient with 28 repeats, 79 in patient with 70 repeats). Neither had a family history of MND nor past medical or family history of psychiatric conditions. The individual with 28 repeats died 2.6 years after symptom onset whereas the individual with 70 repeats was alive 1.6 years after onset.

There was a significant association between *SOD1* carriers and having a family history of MND (*p* = 0.0001) (Table 2). *SOD1* p(.Ile114Thr*)* carriers were studied separately and they were also significantly associated with having a family history of MND (*p* = 0.0003) (Table 2). Family-history details of p(.Ile114Thr) carriers are described in Table 3. The individual with both the *C9orf72* expansion and *SOD1* p(.Ile114Thr) variant was a male who had lower limb-onset ALS age 68 and who developed cognitive impairment as assessed by ECAS. Interestingly, he had no family history of MND or other neurological conditions. He died 65 months (5.4 years) after symptom onset.

**Table 3:** Family histories of *SOD1* p*(*.Ile114Thr*)* carriers

Proband with SOD1 p.I114T variant	Age of onset of proband	Site of onset of proband	Number of affected relatives	Family history of MND details	Other family history
1	61	Limb	1	Father – limb-onset age 63	Parental grandfather – diagnosed with MS
2	64	Limb	3	Sisters × 2, parental cousin – disease site and onset unknown	
3	67	Limb	1	Father – site and onset unknown	Niece – diagnosed with MS
4	57	Limb	2	Father, sister – limb onset	
5	48	Limb	1	Stepsister – site and onset unknown	
6	55	Limb	0	–	Parental grandfather – diagnosed with MS in 40s
7	67	Limb	0	–	
8	68	Limb	0	–	
9	64	Limb	0	–	

*MS* multiple sclerosis

The patient with the *FUS* LoF mutation had young-onset ALS with short survival (20 months from onset) and a family history of MND. The individuals with *NEK1* LoF mutations did not have family histories of MND.

Of those with a family history of MND (28/339, 8.3%), 9/28 (32.1%) had pathogenic *C9orf72* expansions and 6/28 (21.4%) had *SOD1* mutations. Including all pathogenic variants and expansions, there was an overall mutation rate of 16/28, 57.1%. However, even in those without a clear family history of MND the frequency of *C9orf72* was 21/311 (6.8%) and *SOD1* 6/311 (1.9%) with an overall pathogenic mutation rate of 28/311, 9.0%.

## Discussion

### Genetic epidemiology of MND in Scotland

Thirteen per cent of pwMND met criteria for having a pathogenic mutation/expansion. A further 11.2% of the cohort had potential ‘hot’ VUS but there was insufficient evidence to classify these variants as causative and they would not have been reportable clinically. As such, we report a realistic impression of the MND genetic landscape in Scotland. Almost two thirds of those with family history of MND and almost a tenth of those without a family history of MND had a potentially pathogenic mutation. The rates amongst apparently sporadic cases are very similar to those reported in a recent large study of ALS genomes, using a 90-gene panel and ACMG-AMP led classification [8].

The most important regions of interest in this incident Scottish cohort are: the *C9orf72* expansion and the *SOD1* and *NEK1* genes. The proportion of *C9orf72* expansion carriers (8.8%) was lower than in a previous Scottish study (10.2%) [5]. However, the 2015–17 cohort is more unselected than our historical cohort and so more representative. The *C9orf72* expansion is the commonest cause of familial MND affecting 32.1% of cases and 6.8% of apparently sporadic cases. Similar rates have been reported in people of European, USA and Australian origin [8, 31, 32]. *SOD1* mutations were identified in 3.5% of cases overall, but 21.4% of familial cases. This is again lower than in our selected 1989–2014 cohort (5% of all cases, 29% of familial cases) though figures are higher than global population estimates (1–2% sporadic and 12% familial cases [32, 33]). As before, the p.(Ile114Thr) variant is the biggest contributor to this observation, implicated in 17.9% (5/28) of familial cases. The relative ethnic homogeneity of the Scottish MND population is likely a factor in the persistence of this variant [13]. This has significant implications as antisense oligonucleotide (ASO) gene-modifying treatments for *SOD1* carriers appear promising, with the potential to treat up to 4% of the Scottish MND population [9].

We also discovered potentially pathogenic LoF variants in the *NEK1* gene. This gene is as yet poorly characterised in MND populations but loss of function is considered mechanistic [34]. Of note, the *NEK1* missense mutation p.(Arg261His) was also identified in this study (Supplementary Material 2). First identified in an isolated community in the Netherlands, it was thought to be a risk variant for ALS using meta-analysed data (*p* = 4.8 × 10^–5^, OR 2.4 in cases versus controls) [35]. In the Scottish 1989–2014 cohort, five cases and two controls had this variant and it was considered Likely Pathogenic. In the 2015–17 cohort, it was present in nine cases and seven controls. Other clinical samples reported to ClinVar suggest that the variant may be a VUS, Likely Benign or Benign. In the absence of functional studies, we would now classify this variant as likely benign, present in a total of 1.8% of cases and 0.7% of controls in Scottish MND cohorts (1989–2017).

### Genotype–phenotype associations

Univariate association testing of *C9orf72* expansion carriers showed that they have significantly poorer ALS-Specific ECAS scores; this finding parallels other studies and supports a link between *C9orf72* and MND-FTD (Motor Neuron Disease with Frontotemporal Dementia) spectrum disorders [36–39]. This highlights the utility and importance of early cognitive assessment using the ECAS assessment tool following diagnosis of MND. The penetrance for the *C9orf72* expansion is incomplete but is thought to be higher in MND than in pure FTD; it is also unaffected by prior family history of disease and increases with age [40]. Although previous trials of ASO therapies for *C9orf72* expansions were terminated due to lack of efficacy, exploration of other genetically-targeted drug treatments for *C9orf72* expansions are ongoing and an urgent priority for the MND/ALS community. Early identification of cognitive impairment will therefore be crucial to guide appropriate genetic testing and potential drug trial participation before people with *C9orf72* expansions lose capacity. Whilst a family history of MND was significantly associated with having a *C9orf72* expansion, having a family history of young-onset dementia, psychiatric disease or other neurological disease did not reach correct significance (*p* = 0.1, *p* = 0.2, and *p* = 0.03 respectively). This is perhaps surprising [41] but likely reflects the low patient numbers and potential under-reporting of family histories.

Whilst it did not meet Bonferroni-corrected threshold for significance, fewer *C9orf72* expansion carriers in our population were initiated on NIV (*p* = 0.003). *C9orf72* expansion carriers are thought to have fast respiratory decline [42]. We might infer that this population might not have had opportunity to commence on NIV due to inability to consent and comply with treatment (because of cognitive impairment) and because of rapidly progressive disease. Inclusion of Forced Vital Capacity respiratory measures in a longitudinal survival study might help to validate these findings. In the meantime, early assessment of *C9orf72* status and ECAS cognitive assessment in a clinical setting could guide intervention strategies and help to maximise patient access to available intervention. The male-to-female ratio in *C9orf72* expansion carriers was 50:50, ie., more females that would be expected in a typical MND cohort. In a Scottish study, we found that significantly fewer females than males were commenced on NIV (*p* < 0.0001) [13]. This was unexplained but, on reflection, *C9orf72* status might be a contributor.

We identified two patients with intermediate-length repeat expansions who both had classical ALS phenotypes and cognitive impairment. Intermediate repeats are more common in those with neuropsychiatric disease (including FTD) and our results provide further evidence of this phenotype [30].

In contrast to *C9orf72*, more *SOD1* mutation carriers started NIV, though this did not reach corrected significance (*p* = 0.007). This may be due to their having more predictable limb-onset ALS disease. ECAS scores of *SOD1* carriers were reflective of the population as a whole; patients with *SOD1* mutations tend not to have significant cognitive impairment and so this was anticipated [43]. Individuals who had the p.(Ile114Thr) mutation all had limb-onset ALS suggesting that the variant may result in a ‘typical’ *SOD1* phenotype, as has been described in recent meta-analyses [44]. Indeed, none required gastrostomy insertion by the time of censorship (p = 0.03), suggesting that bulbar disease was not a prominent feature. Family histories of individuals with the p.(Ile114Thr) variant revealed histories of limb-onset MND with similar ages to the proband, although details about disease site and onset are missing for some. Family histories of multiple sclerosis were also apparent, perhaps implying either a shared genetic aetiology or a historical misdiagnosis of phenotype.

The individual with MND with the *FUS* LoF variant has young-onset rapid progressive disease, meeting the expected phenotypic profile for this gene [45]. The *NEK1* gene is relatively newly described and genotype–phenotype observations are limited but there is a suggestion in the literature that carriers are more likely to have the flail arm/bibrachial phenotype [46]. Our two *NEK1*-variant carriers, however, had ALS (bulbar onset and limb onset, respectively). Interestingly, we identified one patient with both a *C9orf72* expansion and the *SOD1* p.(Ile114Thr) variant. This individual had a typical age of onset and relatively slow progression, perhaps in-keeping with a *SOD1* phenotype, but had cognitive impairment in line with *C9orf72* phenotype.

### Strengths and limitations

Due to the well-established and robust nature of the CARE-MND platform and the Scottish MND register we have been able to gain a wealth of information about our MND population. The 2015–2017 cohort was the first to be studied within the CARE-MND platform and is comprehensive, with ascertainment of 99% of pwMND in Scotland, using capture-recapture methodology [13]. These data were also unaffected by the coronavirus pandemic. We would therefore anticipate that patients diagnosed subsequently would have similar characteristics and future and ongoing analysis of CARE-MND Register data should support this. However, just over half of the incident MND population (54.8%) contributed to this genetic study. Reasons for not achieving higher ascertainment might include patient choice, limited discussions regarding research options prior to the CARE-MND initiative and rapidly deteriorating disease, meaning that patients were less willing to devote time and efforts to research. Currently, genetic research in Scotland does not offer feedback of results and does not lead to treatment modification and so benefits to patients at an individual level are limited. Allowing for these factors, we consider our recruitment figures to be appropriate and reflective of the generosity of the Scottish MND patient community.

We acknowledge that that we have not confirmed variants identified in the incident cohort using Sanger sequencing. Whilst concordance between next-generation sequencing (NGS) and Sanger techniques is now excellent, the risk of false positives with NGS may be 1.3% [47]. One reason that this was not pursued was that sequencing was performed as part of a research study only, with results not being fed back to patients. In Scotland, all patients with MND are encouraged to store DNA in an NHS clinical-approved laboratory and if a drug were to become available for an MND-associated gene, the patient or their family members could activate confirmatory testing. We have also adopted a panel-sequencing approach for this study to make the results more generalisable to clinical practice. Advances in gene sequencing technology, including long-read sequencing, may allow the detection of even more rare genetic variation in future studies [48].

The number of patients with mutations also is small to make firm conclusions, especially with regard to genotype–phenotype associations. Bonferroni correction of statistical testing gives us conservative estimates which are hypothesis-generating, though near-significant results do confirm clinical observations in practice.

The majority of pwMND in Scotland are of White Scottish origin[13] (98.8% in this study). The *SOD1* p.(Ile114Thr) variant has been detected in European, North American, and Australasian populations but our results may not be generalisable to other ethnic groups or populations.

## Conclusions

Our results show that the CARE-MND database provides a wealth of information about people diagnosed with MND which can be used to inform pwMND and stakeholders. By employing structured variant classification and using an extensive gene-panel approach we have provided a realistic estimate of the frequency of rare variants in the Scottish MND population for the first time. This information has subsequently informed patient information sources. We have confirmed the frequency of a specific SOD1 variant (*SOD1* p.(Ile114Thr)) in the population and have detailed associated phenotypic characteristics; awareness of such key local mutations is essential for the delivery of future genetically-targeted drug trials and drug approvals. *NEK1* LoF variant carriers (0.6%) make up a small but important subset of patients. As a consequence of this study, the Scottish Neurodegenerative Disease Gene Panel has been updated to include *NEK1 (*https://www.nhsggc.org.uk/media/271442/germlinetestdirectory_v10.pdf*)*. As *NEK1* carriers were all apparently sporadic cases, future work into functional and pathological correlates is merited. We have also demonstrated that the *SOD1* p.(Ile114Thr) variant and the *C9orf72* expansion can co-exist and should be tested simultaneously—to our knowledge this is not widely described. However, more systematic gene testing of pwMND would likely reveal further cases and help to determine which gene phenotype is more strongly manifested. From a practical perspective, we suggest that early clinical gene testing may guide management, either by prompting consideration of NIV prior to cognitive decline (*C9orf72*) or by preparing patients early for the likelihood of NIV and/or reduced pressure to consider urgent gastrostomy insertion (*SOD1*).

### Supplementary Information

Below is the link to the electronic supplementary material.

Supplementary file1 (DOCX 31 KB)

Supplementary file2 (DOCX 18 KB)

Supplementary file3 (DOCX 13 KB)

## Data Availability

Data supporting the genetic findings of this study are available within the article and supplementary material. Raw CARE-MND data are not available due to their containing information that could compromise the privacy of research participants. Further information about the CARE-MND database can be found at: https://www.care-mnd.org.uk/.
