# Supplementary material for: Genotypes and phenotypes of motor neuron disease: an update of the genetic landscape in Scotland
Source: J Neurol. 2024 Jun 9;271(8):5256–66. doi: 10.1007/s00415-024-12450-w (PMC11319561; doi:10.1007/s00415-024-12450-w)
Supplement: Supplementary file 1 — Supplementary file1 (DOCX 31 KB) [file 415_2024_12450_MOESM1_ESM.docx]

**Supplementary Material 1: Consensus Methodology for Variant Classification**

Filtered variants were annotated and population frequency filters were applied using VarSeq Golden Helix software[1] to include only those with minor allele frequency (MAF) ≤0.01 in gnomAD 2.0.1v3.[2]

A rodent model of a given mutation was accepted as supportive evidence if the rodent had motor or cognitive symptoms, there was impact on survival, or there was proven loss of motor neurons pathologically[3–5].  Pathologically similar findings without neurodegeneration were not accepted (eg. presence of DP43 mis-localization, dipeptide repeats, *C9of72* foci). The only accepted *in vitro* assay was cytoplasmic localization/nuclear exclusion of a *FUS* variant.

Co-segregation was determined using methods described by Jarvik et al.[6] DNA samples from the Lothian Birth Cohorts (n=1385) were used as ancestry-matched controls to identify variants enriched in cases versus controls as per ACMG-AMP guidelines. The gnomAD database was used as a population control data set.[2] Variants were considered significantly more prevalent in cases versus controls if odds ratio >5.0 and confidence intervals did not cross 1.0 (ACMG-AMP criteria PS4).[7] Additionally, gnomAD was used as a population control data set (ACMG criteria PM2).[2] Variants meeting criteria thresholds for a pathogenic or likely pathogenic classification were reported. Variants of uncertain clinical significance (VUS) that fulfilled some criteria for being pathogenic, without reaching strict ACMG-AMP thresholds for significance, were considered under the Bayes rules outlined by Tavtigian et al to determine a posterior probability of their being potentially pathogenic (probability >0.5).[8] Variants were annotated using multiple in silico prediction algorithms from the Database for Nonsynonymous SNPs and their Functional Predictions (dbNSFP)[9] and included: SIFT, PolyPhen2 HDIV and HVAR, Mutation Taster, Mutation Assessor, FATHMM, PROVEAN, GERP and PhastCons. Measures of impact on splice site included scores derived from adaptive boost (Ada) and random forest (RF) models[10].

**References**

1. Golden Helix I. Golden Helix, Inc, Bozeman, MT. 2018 [cited 2018 Nov 11]. VarSeq. Available from: http://goldenhelix.com

2. Karczewski KJ, Francioli LC, Tiao G, Cummings BB, Alföldi J, Wang Q, et al. The mutational constraint spectrum quantified from variation in 141,456 humans. Nature [Internet]. 2020 May 28;581(7809):434–43. Available from: https://www.nature.com/articles/s41586-020-2308-7

3. Picher-Martel V, Valdmanis PN, Gould P V., Julien JP, Dupré N. From animal models to human disease: a genetic approach for personalized medicine in ALS. Acta Neuropathol Commun [Internet]. 2016 Jul 11 [cited 2017 May 12];4(1):70. Available from: http://www.ncbi.nlm.nih.gov/pubmed/27400686

4. Philips T, Rothstein JD. Rodent Models of Amyotrophic Lateral Sclerosis. In: Current Protocols in Pharmacology [Internet]. Hoboken, NJ, USA: John Wiley & Sons, Inc.; 2015 [cited 2017 Sep 19]. p. 5.67.1-5.67.21. Available from: http://www.ncbi.nlm.nih.gov/pubmed/26344214

5. Nalbandian A, Llewellyn KJ, Badadani M, Yin HZ, Nguyen C, Katheria V, et al. A progressive translational mouse model of human valosin-containing protein disease: the VCP(R155H/+) mouse. Muscle Nerve [Internet]. 2013 Feb [cited 2017 Sep 19];47(2):260–70. Available from: http://doi.wiley.com/10.1002/mus.23522

6. Jarvik GP, Browning BL. Consideration of Cosegregation in the Pathogenicity Classification of Genomic Variants. Am J Hum Genet [Internet]. 2016 Jun 2 [cited 2017 Sep 19];98(6):1077–81. Available from: http://www.ncbi.nlm.nih.gov/pubmed/27236918

7. Richards S, Aziz N, Bale S, Bick D, Das S, Gastier-Foster J, et al. Standards and guidelines for the interpretation of sequence variants: a joint consensus recommendation of the American College of Medical Genetics and Genomics and the Association for Molecular Pathology. Genet Med [Internet]. 2015 May [cited 2016 Sep 1];17(5):405–24. Available from: http://www.ncbi.nlm.nih.gov/pubmed/25741868

8. Tavtigian S V., Greenblatt MS, Harrison SM, Nussbaum RL, Prabhu SA, Boucher KM, et al. Modeling the ACMG/AMP variant classification guidelines as a Bayesian classification framework. Genet Med [Internet]. 2018 Sep;20(9):1054–60. Available from: http://www.nature.com/articles/gim2017210

9. Liu X, Wu C, Li C, Boerwinkle E. dbNSFP v3.0: A One-Stop Database of Functional Predictions and Annotations for Human Nonsynonymous and Splice-Site SNVs. Hum Mutat [Internet]. 2016 Mar [cited 2019 Jul 18];37(3):235–41. Available from: http://www.ncbi.nlm.nih.gov/pubmed/26555599

10. Jian X, Boerwinkle E, Liu X. In silico prediction of splice-altering single nucleotide variants in the human genome. Nucleic Acids Res [Internet]. 2014 Dec 16 [cited 2019 Jul 23];42(22):13534–44. Available from: http://www.ncbi.nlm.nih.gov/pubmed/25416802
