# Supplementary material for: Genotypes and phenotypes of motor neuron disease: an update of the genetic landscape in Scotland
Source: J Neurol. 2024 Jun 9;271(8):5256–66. doi: 10.1007/s00415-024-12450-w (PMC11319561; doi:10.1007/s00415-024-12450-w)
Supplement: Supplementary file 2 — Supplementary file2 (DOCX 18 KB) [file 415_2024_12450_MOESM2_ESM.docx]

**Supplementary Material 2: Variants of uncertain clinical significance identified in incident MND cohort**

| **Gene** | | **Genomic Position** | **Variant DNA Change** | **Variant Protein Change** | **Variant Type** | **Number cases** | **Number controls** |
| --- | --- | --- | --- | --- | --- | --- | --- |
| *ANXA11* | 10:81923309A>T | c.1010T>A | p.Leu337His | Missense | 1 | 0 |  |
| *APP* | 21:27423322T>A | c.656A>T | p.Asp219Val | Missense | 1 | 0 |  |
| *CCNF* | 16:2487264G>A | c.481G>A | p.Gly161Arg | Missense | 1 | 0 |  |
| *CCNF* | 16:2481265G>A | c.151G>A | p.Asp51Asn | Missense | 1 | 0 |  |
| *CSF1R* | 5:149441322C>T | c.1717G>A | p.Glu573Lys | Missense | 1 | 0 |  |
| *DAO* | 12:109283278C>T | c.343C>T | p.Arg115Trp | Missense | 1 | 0 |  |
| *DCTN1* | 2:74605284C>T | c.122G>A | p.Arg41Gln | Missense | 1 | 0 |  |
| *DCTN1* | 2:74595092A>C | c.2015+5G>A |  | Intronic splice site | 1 | 0 |  |
| *DCTN1* | 2:74593111C>T | c.2795G>A | p.Arg932His | Missense | 1 | 0 |  |
| *ERBB4* | 2:212293178T>C | c.2674A>G | p.Ile892Val | Missense | 1 | 0 |  |
| *FUS* | 16:31202343C>T | c.1453C>T | p.Arg485Trp | Missense | 1 | 0 |  |
| *GRN* | 17:42429718T>C | c.1423T>C | p.Cys475Arg | Missense | 1 | 0 |  |
| *GRN* | 17:42429727C>T | c.1432C>T | p.Arg478Cys | Missense | 1 | 0 |  |
| *MAPT* | 17:44055753G>T | c.320G>T | p.Gly107Val | Missense | 1 | 0 |  |
| *MATR3* | 5:138661306C>T | c.2326C>T | p.Pro776Ser | Missense | 1 | 0 |  |
| *NEFH* | 22:29876289->C | c.41dupC | p.Phe15Valfs*83 | LoF frameshift | 1 | 0 |  |
| *NEK1* | 4:170510659A>C | c.403T>G | p.Phe135Val | Missense | 1 | 0 |  |
| *NEK1* | 4:170398485G>A | c.2140C>T | p.Arg714Cys | Missense | 1 | 0 |  |
| *NEK1* | 4:170501992C>G | c.868+1G>C |  | LoF splice donor | 2 | 0 |  |
| *NEK1* | 4:170511956A>G | c.317T>C | p.Leu106Ser | Missense | 1 | 0 |  |
| *NOTCH3* | 19:15272414G>A | c.6025C>T | p.Arg2009Trp | Missense | 1 | 0 |  |
| *NOTCH3* | 19:15298776C>T | c.1522G>A | p.Val508Met | Missense | 1 | 0 |  |
| *NOTCH3* | 19:15288789G>C | c.3950C>G | p.Pro1317Arg | Missense | 1 | 0 |  |
| *NOTCH3* | 19:15302615C>G | c.743G>C | p.Gly248Ala | Missense | 1 | 3 |  |
| *OPTN* | 10:13178846G>A | c.1714G>A | p.Val572Met | Missense | 1 | 0 |  |
| *PRPH* | 12:49691776C>T | c.1303C>T | p.Arg435Trp | Missense | 1 | 0 |  |
| *PSEN1* | 14:73640329T>G | c.394T>G | p.Ser132Ala | Missense | 1 | 0 |  |
| *PSEN1* | 1:227073265C>T | c.383C>T | p.Thr128Ile | Missense | 1 | 1 |  |
| *SETX* | 9:135203653A>C | c.3332T>G | p.Leu1111Trp | Missense | 1 | 0 |  |
| *SQSTM1* | 5:179250993C>T | c.437C>T | p.Pro146Leu | Missense | 1 | 0 |  |
| *SQSTM1* | 5:179260077G>A | c.800G>A | p.Arg267His | Missense | 2 | 0 |  |
| *TBK1* | 12:64889529A>C | c.1694A>C | p.Gln565Pro | Missense | 1 | 0 |  |
| *VCP* | 9:35065361G>A | c.463C>T | p.Arg155Cys | Missense | 1 | 0 |  |
| ***Heterozygous Variants in Recessive Genes*** | | | | | | | |
| *ALS2* | *2:202614512C>T* | *c.1738G>A* | *p.Val580Ile* | *Missense* | *1* | *0* |  |
| *ALS2* | *2:202574703T>C* | *c.4181A>G* | *p.Tyr1394Cys* | *Missense* | *1* | *0* |  |
| *ALS2* | *2:202626404C>T* | *c.313G>A* | *p.Ala105Thr* | *Missense* | *1* | *0* |  |
| *BSCL2* | *11:62458810A>C* | *c.755T>G* | *p.Phe252Cys* | *Missense* | *1* | *0* |  |
| *HTRA1* | *10:124266357C>T* | *c.928C>T* | *p.Arg310Cys* | *Missense* | *1* | *0* |  |
| *SPG11* | *15:44949428AT/-* | *c.733_734delAT* | *p.Met245Valfs*2* | *LoF frameshift* | *1* | *0* |  |
| *SPG11* | *15:44855329A>G* | *c.7322T>C* | *p.Leu2441Pro* | *Missense* | *1* | *0* |  |
| *SPG11* | *15:44925771A>G* | *c.1667T>C* | *p.Phe556Ser* | *Missense* | *1* | *0* |  |
| *SPG11* | *15:44876486C>T* | *c.5392G>A* | *p.Glu1798Lys* | *Missense* | *1* | *0* |  |
