# Supplementary material for: Genotypes and phenotypes of motor neuron disease: an update of the genetic landscape in Scotland
Source: J Neurol. 2024 Jun 9;271(8):5256–66. doi: 10.1007/s00415-024-12450-w (PMC11319561; doi:10.1007/s00415-024-12450-w)
Supplement: Supplementary file 3 — Supplementary file3 (DOCX 13 KB) [file 415_2024_12450_MOESM3_ESM.docx]

**Supplementary Material 3: Digenic Variant Cases**

| **Gene 1** | **Variant Protein Change 1** | **Gene 2** | **Variant Protein Change 2** |
| --- | --- | --- | --- |
| *C9orf72* | Expansion | *SOD1* | p.Ile114Thr |
| *C9orf72* | Expansion | *ALS2* | p.Val580Ile |
| *SOD1* | p.Ala146Asp | *SETX* | p.Leu1111Trp |
| *MATR3* | p.Pro776Ser | *SQSTM1* | p.Arg267His |
| *NEK1* | p.Arg714Cys | *DCTN1* | p.Arg932His |
| *FUS* | p.Tyr479Metfs*50 | *SPG11* | p.Glu1798Lys |
| *ALS2* | p.Ala105Thr | *NOTCH3* | p.Pro1317Arg |
